# Building pan-genome infrastructures for crop plants and their use in association genetics

**DOI:** 10.1093/dnares/dsaa030

**Published:** 2021-01-22

**Authors:** Murukarthick Jayakodi, Mona Schreiber, Nils Stein, Martin Mascher

**Affiliations:** 1 Department of Genebank, Leibniz Institute of Plant Genetics and Crop Plant Research (IPK) Gatersleben, Seeland, Germany; 2 Center for Integrated Breeding Research (CiBreed), Georg-August-University Göttingen, Göttingen, Germany; 3 German Centre for Integrative Biodiversity Research (iDiv) Halle-Jena-Leipzig, Leipzig, Saxony, Germany

**Keywords:** genomics, pan-genome, crop plants, association genetics, genome sequencing

## Abstract

Pan-genomic studies aim at representing the entire sequence diversity within a species to provide useful resources for evolutionary studies, functional genomics and breeding of cultivated plants. Cost reductions in high-throughput sequencing and advances in sequence assembly algorithms have made it possible to create multiple reference genomes along with a catalogue of all forms of genetic variations in plant species with large and complex or polyploid genomes. In this review, we summarize the current approaches to building pan-genomes as an *in silico* representation of plant sequence diversity and outline relevant methods for their effective utilization in linking structural with phenotypic variation. We propose as future research avenues (i) transcriptomic and epigenomic studies across multiple reference genomes and (ii) the development of user-friendly and feature-rich pan-genome browsers.

## 1. The pan-genome concept 

Crop species exhibit extensive phenotypic variation in agronomic characters, such as phenology, yield, metabolite biosynthesis and response to biotic and abiotic stresses. Effective utilization of genetic variation is key to crop improvement to meet future challenges of climate change and evolving pathogens.^1–3^ DNA sequence polymorphisms are commonly classified into single-nucleotide polymorphisms (SNPs), short insertions and deletions (indels) and larger (>50 bp) structural variations (SVs), which comprise presence/absence variants (PAVs) and copy number variants (CNVs) as well as balanced rearrangements, namely inversions and inter/intra-chromosomal translocations.^4^^,^^5^ Capturing the full spectrum of natural SV in a species is challenging. In the past decade, reference genome sequence assemblies and catalogues of sequence diversity were generated for many crop species, among them the major cereal^6–9^ and legume crops.^10^^,^^11^ These projects assembled genome sequences for a single genotype and detected SNPs and indels from high-throughput sequencing data mapped to the reference genome sequence. Although a single reference genome sequence is the backbone of a genomic infrastructure, it cannot represent the full complement of sequence diversity of a species. Especially challenging are large-structural variants that are difficult to capture by short-read sequencing and reference-based analysis. Nevertheless, several studies have shown that this class of variants can play a vital role in determining agronomic traits,^12–15^ local adaptation and speciation.^16–20^

The concept of a ‘pan-genome’ refers to the universe of genome sequences existing in a species. Representing each and every sequence variant segregating in the pan-genome is a distant goal. First-generation pan-genome studies commonly aimed at discovering as many structural variants as possible with a diverse, but necessarily limited set of genotypes. Pan-genomic studies have been conducted in various model and crop plants including *Arabidopsis thaliana*,^21^^,^^22^*Brachypodium distachyon*,^23^*Brassica oleracea*,^24^ tomato,^25^ rice,^26–28^ soybean,^29^ rapeseed,^30^ wheat^31^ and barley.^32^

To date, the pan-genome concept has been discussed extensively regarding definitions, approaches, computational challenges and potential applications.^33–39^ Moreover, the development of computational tools for pan-genome representations and visualizations have already been discussed in detail elsewhere.^35^^,^^40–42^ Here, we review strategies for (i) building pan-genomes from reference-quality genome sequence assemblies, (ii) genotyping SVs discovered in large diversity panels using short-read resequencing and (iii) linking SVs to phenotypes in genome-wide association studies (GWAS). We propose transcriptomic and epigenomic studies focusing on accessions with high-quality genome assemblies as well as the development of pan-genome visualization solutions (e.g. web browser) as future research avenue.

## 2. Selecting germplasm for a sequence assembly

**Figure 1:**
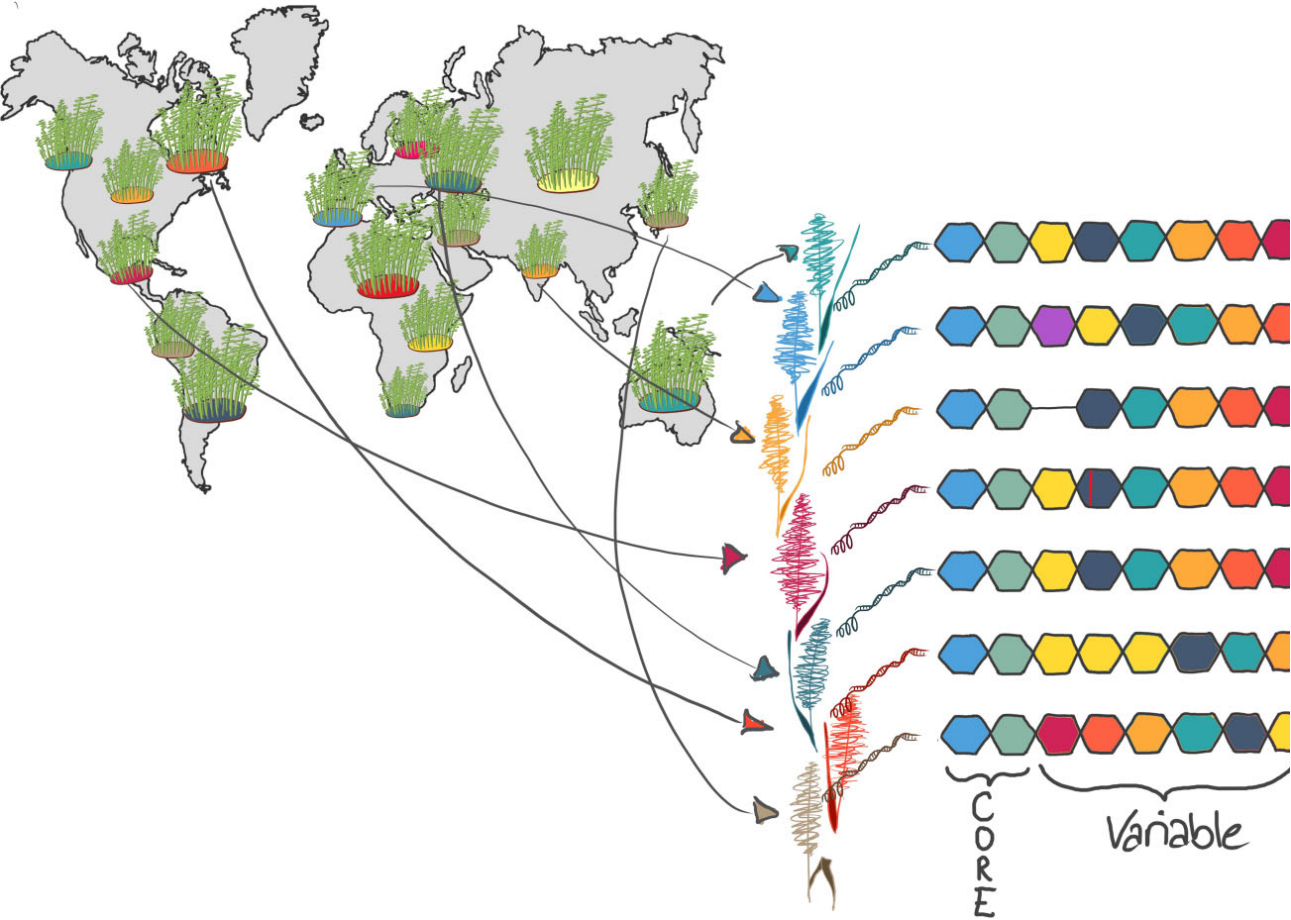
Pan-genome selection and construction. Representative genotypes are chosen from genetically diverse populations based on genome-wide genotypic data for *ex situ* germplasm collections. Chromosome-scale genome assemblies are built for a small, but representative core set. The pan-genome compartments such as core (i.e. genomic sequences present in all individual of a species) and variable (i.e. sequences found in some/few individuals) are identified from the *de novo* assemblies.

**Figure 2:**
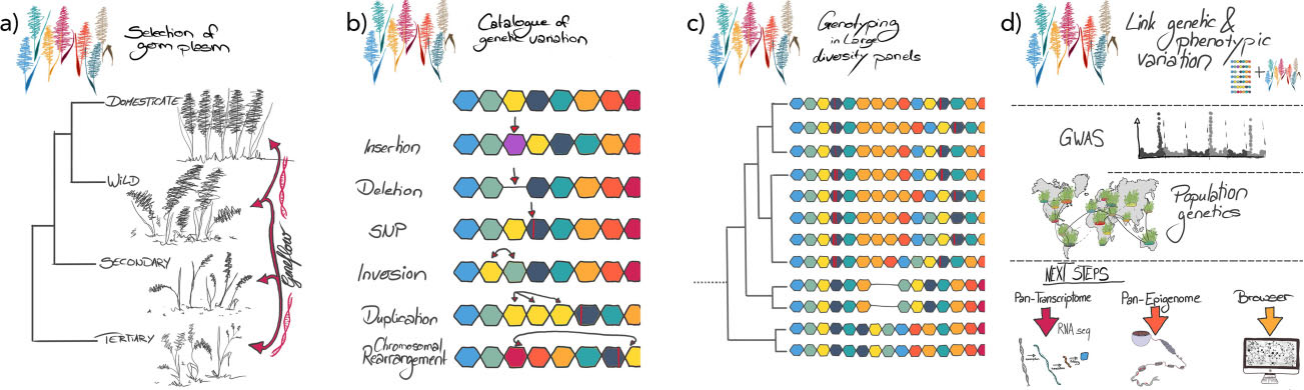
A pan-genome workflow. (a) A representative core set of accessions is selected from the domesticated and wild gene pools. Accessions from secondary and tertiary gene pools are added to build the pan-genome at genus level. (b) Reference-quality genomes (represented in coloured hexagons) are generated for a small set of accessions and aligned to each other to catalogue the small, medium and large variants (SVs) including insertion, deletion, inversion and translocation. (c) Binary SVs (large insertions and deletions) are genotyped (Fig. 3 for genotyping strategy) in a wider panel of germplasm using short-read sequencing. Each hexagon order represents individual genome from distinct accessions. (d) A combination of assemblies and resequencing data underpins genetic analyses such as GWAS and population genetic inquiries into pan-genome complexity. Accessory functional data on gene expression and gene profiles will decorate pan-genomes to assist hypothesis generation. All information is provided to research community in a user-friendly web interface (browser).

The first step in setting up a pan-genome infrastructure is the selection of a diverse set of representative genotypes for sequence assembly (Fig. 1). The goal is to capture as many genetic variants as possible with a limited panel of genotypes. Genebanks, i.e. national or international germplasm repositories, host hundreds to thousands of accessions of all major crop species, but minor crops might be not as well represented in *ex situ* collections (http://www.fao.org/3/i1500e/i1500e00.htm). Genome-wide genotypic data for entire genebank collections or representative subsets are crucial to select diverse accessions covering all major germplasm groups in a species. Such genebank genomics studies have been reported recently for barley,^43^ wheat,^44^ maize^45^ and rice.^46^ Genotyping-by-sequencing (GBS)^47^ was used to fingerprint more than 20,000 wild and domesticated barleys^43^ from the German *ex situ* genebank. Researchers from the International Maize and Wheat Improvement Centre (CIMMYT) report GBS profiles for 44,624 wheat lines from the breeding programs^44^^,^^48^ as well as DArTseq data for 80,000 wheat accessions from the genebanks of CIMMYT and the International Centre for Agricultural Research in the Dry Areas. The genomes of more than 3000 cultivated rice accessions from the International Rice Research Institute genebank were sequenced to generate a digital genebank and a pan-genome.^46^ There are various approaches for selecting coresets.^49^ For example, the tool Corehunter^50^ implements different algorithms operating on genetic distance matrices to maximize diversity, representativeness and/or allelic richness of core sets. Custom selections may also be made from clustering the diversity space as represented by principle component analysis^51^ or model-based ancestry estimation.^52^ Pan-genome panels may include domesticated accessions as well as accessions of conspecific wild progenitors or ancestors of polyploid species, e.g. maize and teosinte or wheat and wild emmer and *Aegilops tauschii.* Crop-wild relatives in the secondary and tertiary gene pools^53^ may be included to serve as out-groups, e.g. to determine ancestral states for SVs (Fig. 2), or because of their relevance in introgression breeding. In addition to focusing on maximizing representativeness of global diversity in a crop, a pan-genome project may also select genotypes that have played an important role in breeding and genetics such as founder genotypes of breeding programs, parents of experimental populations^54^ or genotypes amenable to genetic transformation^55^^,^^56^ may be included to maximize the benefits for the research and breeding community. Vice versa, the accessions included in pan-genomic studies are poised to become reference genotypes in future genetic and functional studies by virtue of the genomic resources associated with them.

## 3. Moving from short-read resequencing to long-read reference genomes

### 3.1. Alignment vs. assembly

High-throughput short-read sequencing on the Illumina platform has been extensively used for plant genome assembly, population genomics and GWAS studies, but it has important drawbacks. The intergenic space in plant genomes is mainly derived from transposable elements (TEs).^57^ Since Illumina reads are only up to a few hundred basepairs in length, they cannot traverse most repeats, leading to fragmented and incomplete genome assemblies. Similarly, applying short-read sequencing data to detect SVs using read depth or paired-end information (‘split reads’) is prone to errors in very complex regions, such as plant resistance gene loci. Alignment of long (>10 kb) reads to a reference genome can overcome some of these challenges. Still, even with long reads, insertions exceeding the read length, tandem and segmental duplications,^58^^,^^59^ as well as balanced events such as large inversions (>1 Mb),^60–62^ are challenging to detect from alignments to a single reference genome.

### 3.2. Assembly methods

*De novo* assembly of multiple high-quality reference genome sequences and their comparison by pair-wise sequence alignment is arguably the most powerful and accurate approach to detect all types of sequence variant at base-level resolution.^62^ The progress in genome sequencing and assembly methods in the past two decades has been tremendous. The first approaches at whole-genome assembly, namely hierarchical sequencing of bacterial artificial chromosomes on the Sanger platform could only be implemented by international consortia even for small-sized genomes like *Arabidopsis*^63^ or rice.^9^ The development of high-throughput short-read sequencing first on the 454, then on the Illumina platforms,^64^ enabled the generation of draft genomes for many plant sequences, including most crops.^65^^,^^66^ But still assembly contiguous genome sequences from short-reads was a complicated and resource-intensive task^67^^,^^68^ and did not scale well to tens to hundreds of genomes. Multiple short-read libraries with various insert sizes were required for scaffolding contig-level assemblies that were often too fragmented to be useful on their own. Complementary approaches such as optical mapping,^69^ genetic mapping^70^ and chromosome conformation capture sequencing (Hi-C)^71^^,^^72^ were required to increase sequence contiguity from kilobase-sized contigs to full chromosomes. Long-read sequencing on the PacBio^73^ and Oxford Nanopore^74^ platforms have conceptually simplified this approach as assembly of long (> 10 kb) reads result in megabase-sized scaffolds even in complex genomes.^75^ Yet, the high error rate of long-read sequencing (10–15%) requires substantial computational resource for correction and overlap determination—to a degree that assembly of polyploid plant genome could take months.^76^ The need for vast computational resource to assemble large (>1 Gb) plant genomes has recently been obviated by the development of accurate long-sequencing on the PacBio platform.^77^ Repeated read-out of the same DNA fragment by circular consensus sequencing yield reads in the 15–25 kb range with error rate below 1%.^76^ State-of-the art algorithms (HiCanu^78^ and hifiasm^79^) can now assemble human-sized genomes to megabase-scale contiguity within hours on standard compute servers.

### 3.3. Assembly approaches for pan-genomics

We predict that accurate long-read sequencing is a breakthrough technology that will greatly improve our ability to assemble large and complex, heterozygous or polyploid genomes and to do this in timeframe that enabling scaling to pan-genomes. Highly contiguous and accurate genome assemblies will provide access to regions previously inaccessible to sequence analysis such as centromeres^80^ or loci involved in response to pathogens.^81^^,^^82^ However, it should also be kept in mind that any genome assembly can contain errors potentially giving rise to spurious SV calls.^62^^,^^83^ Complementary evidence provided by independent mapping approaches, such as optical maps and Hi-C, are needed to validate and correct assemblies to increase confidence in structural variant calls, particularly for reciprocal events such as inversions and translocations.

At the time of writing, it is an ambitious, but not unrealistic research goal to generate tens of high-quality reference genomes for large-genome plant species and hundreds of reference genomes for smaller species within the timeframe of 1 year. In plants, whole-genome assembly-based pan-genomes have been reported for rice (number of accessions, *n* = 16),^28^^,^^46^^,^^84^ barley (*n* = 20),^32^ wheat (*n* = 10),^31^ maize [*n* = 26; NAM Genomes Project (https://nam-genomes.org)], *Brachypodium distachyon* (*n* = 54),^23^*Glycine soja* (*n* = 7),^29^*Brassica napus* (*n* = 8)^30^ and soybean (*n* = 26).^85^ Computational method development has focused on fast algorithms for aligning long-reads to reference genomes and reference genomes to each other as well as to call variants from such alignments.^38^ Likewise, genome assembly software has kept pace with methodological advances in long-read sequencing.^78^^,^^79^ Nevertheless, sequence assembly of complex plant genomes remains challenging: algorithms struggle with resolving multiple haplotypes in heterozygous or autopolyploid genomes.^79^^,^^86^ Assemblies might result in fragmented sequences, produce chimeric contigs joining different haplotypes or ignore alternative haplotypes. Even when haploid genome assemblies can be constructed from rare inbred or haploid genotypes in otherwise outcrossing or polypoid species,^87^ detecting and phasing heterozygous SVs remains challenging in these species.

## 4. Constructing an *in silico* representation of the pan-genome

### 4.1. Pan-genome graphs

Once genome sequence assemblies of a diversity panel have been obtained, a common first analysis is to compartmentalize the assembled sequences into the core and the variable genome (Fig. 1). The variable genome comprises sequences that are present in some genotypes, but absent from others. The core genome is present in all individuals of a species and may comprise sequence whose loss is incompatible with proper organismal functioning such as house-keeping genes.^88^ In bacteria, where the pan-genome concept was developed first,^88^ the core and variable compartments refer only to gene sequences. As bacterial genomes are small and mainly composed of coding sequence, this approach is correct and straightforward to implement because methods to cluster genes into orthologous groups are well established. In plant and animals, however, a purely gene-based analysis would ignore a large proportion of diversity present in intergenic sequences. As a consequence of the frequent movement of repetitive elements,^89^ much of the variable component of a plant pan-genome is intergenic and derived from TEs. Since orthologous relationships are hard to establish between copies of TEs in different genotypes, recording all sequence alignments between repetitive elements would result in a data structure of inextricable complexity.

Toolkits for the construction, analysis and visualization of graph-based pan-genomes such as vg toolkit,^42^ minigraph^90^ the Practical Haplotype Graph^91^ are under active development.^40^ As of now, further evaluation and development of heuristics for pruning complex regions is needed before these approaches can be deployed on collections of tens to hundreds of plant genome assemblies in the same standardized and streamlined way as toolkits for SNP genotyping operate on short-read data.^92^^,^^93^ In the meantime, different *ad hoc* approaches have been devised to focus on low-copy, but not necessarily genic, regions. In rapeseed, a pan-genome sequence was constructed by adding the PAV sequences from multiple individual genomes to one single reference genome.^30^ In soybean, a graph-based pan-genome construction was performed with non-redundant SVs against a reference genome.^85^

### 4.2. The single-copy pan-genome

Recently in barley, a so-called ‘single-copy pan-genome’ was built by clustering single-copy regions extracted from multiple chromosome-scale sequence assemblies. This work-around enabled quantitative estimates of pan-genome complexity, such as saturation analysis, and provided a reference to derive bi-allelic SV markers for use in association genetics. However, approaches targeting single-copy regions may prove ineffective in polyploids where even highly conserved house-keeping genes occur in multiple copies in the subgenomes. Moreover, as genic regions are under stronger selective pressure and have reduced sequence diversity, gene-based analyses may underestimate pan-genome complexity. For instance, the gene-based pan-genome of soybean reached a plateau with 25 representative accessions,^85^ but this picture could change entire genomes are considered.

## 5. Genotyping SV in short-read data for association genetics

### 5.1. Need for genotyping SV in larger germplasm panels

Despite continuous methodological advances and cost reductions in the past decade, sequence assembly is still substantially more expensive than resequencing. In large-genome plants species, the size of germplasm panels that can be subjected to *de novo* sequence assembly may not be large enough for GWAS or population genomic analysis. One possible approach for including structural variants into genetic analysis is the use of linked SNPs as proxies. But, several studies have shown that the rapid decay of linkage disequilibrium can result in many SVs that are not tagged by near-by SNPs.^15^^,^^94^ A further conceptual drawback is that even if linked SNPs can pinpoint loci in association scans, causal variants residing in SVs whose sequence is absent from the reference genome would be inaccessible.

### 5.2. Graph-based methods

**Figure 3:**
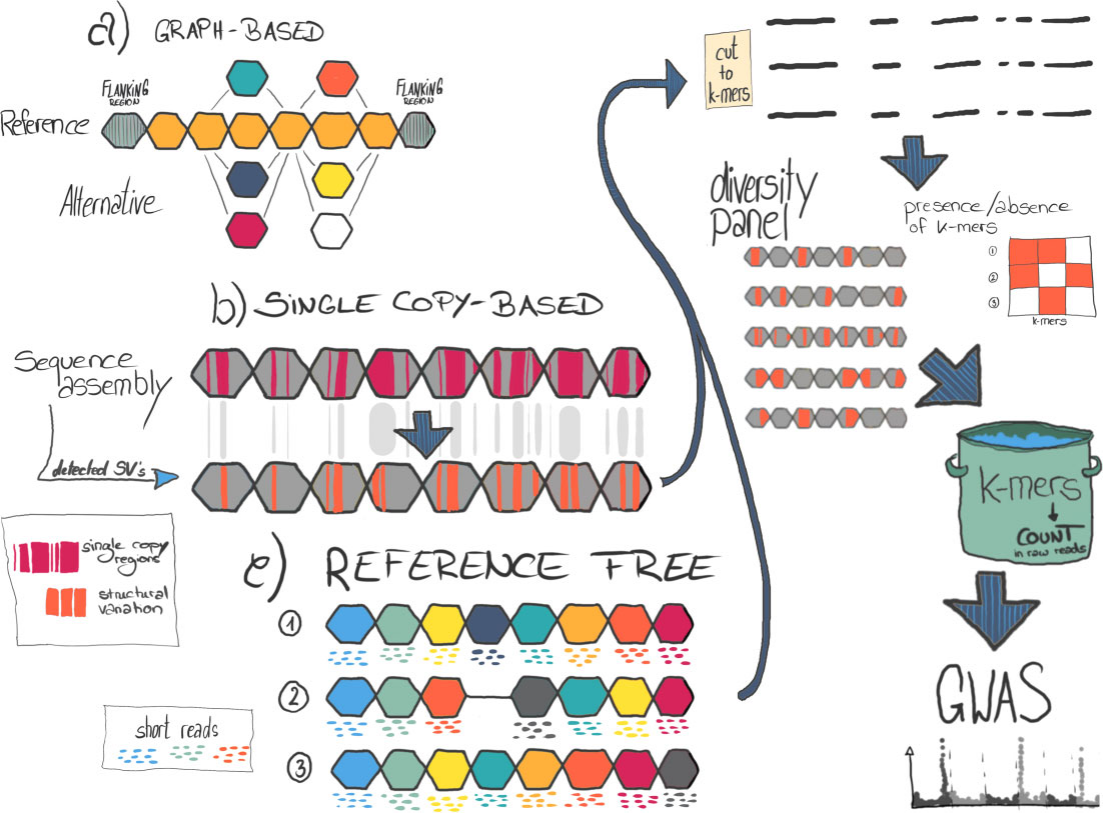
Pan-genome representation and GWAS with SV. (a) A pan-genome graph is constructed from the alignment of chromosome-scale sequence assemblies. This graph represents all types of genetic variants. Sections of the genome are shown as coloured hexagons. Each colour represent one genotypes. SV are represented by different paths through the graph. Tools for constructing and working with pan-genome graphs under active development. Two alternative approaches to capture pan-genomic information in genetic analyses are currently being used. (b) SVs between these genomes are detected from alignments against a common reference genome. Single-copy regions are extracted from the assemblies (mauve colour) and overlapped with SV (orange colour). Single-copy *k*-mers residing in SVs are extracted and their abundance is ascertained in short-read data from a diversity panel to genotype the underlying SV. (c) Reference-free approaches select *k*-mers directly from short-read data of a diversity panel without the need of genome assemblies. Matrices of *k*-mer counts from either single-copy or reference-free approaches are used as markers in GWAS.

Low-coverage whole-genome shotgun sequencing can scale to panels comprising thousands of accessions. Thus, it can complement catalogues of SVs seeded with genome sequence assemblies to discover new, or genotype known events. There are several approaches for genotyping SVs (Fig. 3), which are discovered in a smaller discovery panel, in short-read data for more individuals. One of them is to build variations graphs from SVs discovered in the reference panel (Fig. 3a) and aligning short-reads to the graph.^42^^,^^95–97^ Graph-based SV genotyping requires high read coverage (∼10–30X) to achieve good accuracy.^42^ The advantages of high read depth need to be weighed against larger panel size affording greater statistical power. An alternative approach is to extract defined short sequences (*k*-mers) that are diagnostic for the presence or absence of SV and whose presence can be confidently ascertained in short (< 300 bp) read data. For instance, multiple short *k*-mers with lengths typically in the range of 30–100 bp can be extracted from SVs and queried in short-read resequencing data. Multiple *k*-mers might be combined to increase specificity, mitigate the effects of missing data in low-coverage data and differentiate between different haplotypes sharing the same SV. Choosing *k*-mers from single- or low-copy regions is needed to avoid unspecific matches (Fig. 3b). Single-copy regions do not only comprise genes, but also non-coding regulatory regions and unique TE insertion sites.^98^ Thus, they can serve as anchor points for larger haplotypes even in repetitive regions. Presence/absence tables of the diagnostic *k*-mers act as biallelic marker matrices for use in genetic mapping applications, i.e. GWAS or quantitative trait locus (QTL) mapping in biparental populations. As there are fewer SVs than SNPs, commonly used GWAS methods developed for SNP genotyping or sequencing studies (such as GEMMA^99^ or GAPIT^100^) are readily applicable. As a proof-of-principle, Jayakodi et al.^32^ queried single-copy *k*-mers from structural variants detected in 20 barley assemblies in GBS and WGS data of diversity panels and used a *k*-mer abundance matrix in GWAS scans for morphological characters with a simple genetic architecture. Song et al.^30^ used GWAS with PAV-derived markers to identify SVs associated with silique length, seed weight and flowering time in rapeseed.

### 5.3. Reference-free methods

A conceptually similar *k*-mer-based approach is reference-free association mapping with *k*-mer counts determined only from short read data without any sequence assemblies (Fig. 3c). Instead of diagnostic *k*-mers ascertained from a discovery panel of reference genomes, all *k*-mers occurring in a collection of short reads are catalogued and their presence/absence in individual genotypes is tabulated. As the number of distinct *k*-mers is on the order of billions in large plant genomes, a pre-selection of informative markers is needed for GWAS scans that test for significant marker-trait associations with linear models. Two approaches for *k*-mer-based GWAS in plants have been described. AgRenSeq^101^ combines resistance* (R)* gene enrichment sequencing with fast *k*-mer counting and GWAS scans using general linear models accounting for population structure. Due to the pre-selection of resistance orthologues, AgRenSeq is geared toward the discovery of R genes associated with specific diseases. The kmerGWAS^102^ pipeline first quantifies *k*-mers in either whole genome shotgun or reduced representation sequencing data and then selects a prioritized set of *k*-mers based on a simple and fast statistical test. This smaller set of markers is used in a linear mixed model GWAS accounting for kinship. Both AgRenseq and kmerGWAS do not require a reference genome, but can benefit from it by aligning associated *k*-mers to it to determine chromosomal locations of GWAS peaks. In the absence of a reference genome or a sequence assembly representing the haplotype of interest, *de novo* assembly of reads containing *k*-mers associated with phenotypes may result in complete genes. However, because of the small size of the assembled contigs in the range of 1–10 kb, genomic contextualization is lacking, which could complicate the differentiation between linked and causal variants, in particular, if they reside in intergenic regions for which low-copy informative *k*-mer may be lacking.

As sequence assemblies for more genotypes become available, the pan-genome saturates, that is, the available reference genomes capture most haplotypes segregating at a certain minimum frequency (e.g. 1%) in the population. Then, both reference-agnostic *k*-mer GWAS followed by aligning peak markers to multiple sequence assemblies and GWAS with diagnostic *k*-mers tagging pre-defined haplotypes would conceptually converge. Future work should focus on defining best practices for compiling discovery panels (i.e. high-quality reference genomes), choosing sequencing depth and selecting the most appropriate analysis strategies.

## 6. Beyond the pan-genome

### 6.1. Pan-transcriptomes

SVs can influence gene expression in various ways, for instance by disrupting gene structures, by altering gene copy number or by changing the composition or positioning of *cis*-regulatory sequences.^59^^,^^85^^,^^103^^,^^104^ In addition to changing DNA sequence, SV could affect gene expression by altering epigenomic marks. Unravelling the functional consequences of a given SV, e.g. one associated with an agronomic phenotype, can be challenging. A notable example is a 13 Mb inversion (Inv4m) on maize chromosome 4 that is associated with early flowering.^105^ Expression analysis in more than 430 RNA samples from near-isogenic lines did not reveal one single variant as a convincing causal candidate. Precise perturbations by gene editing or even flipping the inverted haplotype back to the ancestral configuration are possible,^106^ but technically demanding, strategies toward understanding how this inversion altered flowering time. Gene expression atlases across the development of a single genotype have been developed in many plant species^107^^,^^108^ and are recognized as valuable community resources that inform about when, where and how strongly a gene is expressed.

### 6.2. Pan-epigenomes

In the same way, we envision that profiling gene expression and epigenomic marks across a set of genotypes for which chromosome-scale reference genome sequences have been assembled will yield pan-transcriptome and pan-epigenome atlases as permanent community resources. Large-scale expression profiling and population-scale epigenomic studies have been done before, but in the absence of multiple sequence assemblies, data were mapped to a single reference. By integrative analysis of matching genomic, transcriptomic and epigenomic data, it will be possible to analyse the co-location of structural variants and epigenomic variants and gene expression differences between accessions. Such data can help prioritize variants in GWAS studies and guide the development of hypothesis for approaches targeting individual variants (Fig. 2). Recent reports have reported first results in these directions: in tomato, almost, half of the SVs detected in a pan-genome constructed from 14 sequence assemblies overlap with genes and/or flanking regulatory sequences and many of them showed subtle, yet significant changes in gene expression.^59^ In soybean, more than 1,000 SVs were associated with expression changes, notably a candidate gene for iron uptake was identified with RNA-seq evidences.^85^ Yang et al. reported 207 *cis* expression QTLs linked to SVs. Among these, 70 were found to form chromatin loops coding genes in Chromatin Interaction Analysis by Paired-End Tag Sequencing.^103^

### 6.3. Browsers

As methods for sequence assembly and comparative analyses improve, previously inaccessible genomic variants become amenable to genetic study. An outstanding challenge is to make new and more complex data structures such as non-linear graph-based pan-genomes accessible to researchers and breeders who are inexperienced in using command-line tools. An integrated pan-genome browser capable of representing SNPs and large SVs in multi-reference coordinate system, together with their annotations, accessory transcriptomic and epigenomics datasets, as well as links to germplasm repositories would serve as a one-stop shop for genome analysis. However, before this vision can be realized, many obstacles need to be overcome. Among them are the construction of and sequence alignment to pan-genome graphs (e.g. by using vg^42^ or minigraph^90^) as well as merging and consolidating gene annotations across a large and potentially growing number of sequence assemblies.^109–111^ As a first step in this direction, we propose the implementation of web-based tools to query and analyse multiple chromosome-scale reference genomes in a gene-centric manner. The framework needs to include query forms to retrieve allelic gene sequences from multiple reference genomes, inspect multiple-sequence alignments of alleles of genes or larger haplotypes and query the presence of alleles or haplotypes in a wider set of germplasm.

## 7. Concluding remarks and future perspectives

Recent pan-genomic studies have revealed exciting insights into crop domestication and the genetic basis of agronomic traits. We expect, while the analysis and visualization methods mature, pan-genomics will establish as indispensable component in the genomics toolbox of plant geneticists and breeders. Since workflows for sequence assemblies and association genetics are in place, future studies will extend analysis and visualization methods in population genetics, gene expression and epigenetics to the scale of pan-genomes. We anticipate that pan-genomes will become an essential component in studying the diversity of crops and their wild relatives and in developing efficient concepts for their usage in pre-breeding. Digital genebanks based on sequence-based genotyping are feasible right now.^13^^,^^43^ The long-term goals of having genome assemblies for all genebank accessions^112^ is still a distant goal, which, however, has just come a bit closer with the recent breakthroughs in assembly methodology.

## Author contributions

M.J., N.S. and M.M. wrote the paper. M.S. designed and drew figures.

## Funding

The authors’ barley pan-genome research was supported by the German Federal Ministry of Research and Education (BMBF) in frame of the SHAPE II grant to N.S. and M.M. (FKZ 031B0884A).

## Conflict of interest

None declared. 

## References

[1] Esquinas-Alcázar J. 2005, Science and society: protecting crop genetic diversity for food security: political, ethical and technical challenges, Nat. Rev. Genet., 6, 946–53. 16341075 10.1038/nrg1729

[2] Dempewolf H. , Bordoni P., Rieseberg L.H., et al 2010, Food security: crop species diversity, Science, 328, 169–70. 10.1126/science.328.5975.169-e 20378793

[3] Godfray H.C.J. , Beddington J.R., Crute I.R., et al 2010, Food security: the challenge of feeding 9 billion people, Science, 327, 812–8. 20110467 10.1126/science.1185383

[4] Ho S.S. , Urban A.E., Mills R.E. 2020, Structural variation in the sequencing era, Nat. Rev. Genet., 21, 171–89. 31729472 10.1038/s41576-019-0180-9 PMC7402362

[5] Mérot C. , Oomen R.A., Tigano A., et al 2020, A roadmap for understanding the evolutionary significance of structural genomic variation, Trends Ecol. Evol., 35, 561–72. 32521241 10.1016/j.tree.2020.03.002

[6] Mascher M. , Gundlach H., Himmelbach A., et al 2017, A chromosome conformation capture ordered sequence of the barley genome, Nature, 544, 427–33. 28447635 10.1038/nature22043

[7] The International Wheat Genome Sequencing Consortium, 2018, Shifting the limits in wheat research and breeding using a fully annotated reference genome, Science, 361, eaar7191. 30115783 10.1126/science.aar7191

[8] Chandler V.L. , Brendel V. 2002, The maize genome sequencing project, Plant Physiol., 130, 1594–7. 12481042 10.1104/pp.015594 PMC1540264

[9] International Rice Genome Sequencing Project. 2005, The map-based sequence of the rice genome, Nature, 436, 793–800. 16100779 10.1038/nature03895

[10] VandenBosch K.A. , Stacey G. 2003, Summaries of legume genomics projects from around the globe. Community resources for crops and models, Plant Physiol., 131, 840–65. 12644637 10.1104/pp.103.020388 PMC1540284

[11] Varshney R.K. , Close T.J., Singh N.K., et al 2009, Orphan legume crops enter the genomics era!, Curr. Opin. Plant Biol., 12, 202–10. 19157958 10.1016/j.pbi.2008.12.004

[12] Saxena R.K. , Edwards D., Varshney R.K., 2014, Structural variations in plant genomes, Brief Funct. Genom., 13, 296–307. 10.1093/bfgp/elu016 PMC4110416 24907366

[13] Fuentes R.R. , Chebotarov D., Duitama J., et al 2019, Structural variants in 3000 rice genomes, Genome Res., 29, 870–80. 30992303 10.1101/gr.241240.118 PMC6499320

[14] Zhang Z. , Mao L., Chen H., et al 2015, Genome-wide mapping of structural variations reveals a copy number variant that determines reproductive morphology in cucumber, Plant Cell., 27, 1595–604. 26002866 10.1105/tpc.114.135848 PMC4498199

[15] Zhou Y. , Minio A., Massonnet M., et al 2019, The population genetics of structural variants in grapevine domestication, Nat. Plants, 5, 965–79. 31506640 10.1038/s41477-019-0507-8

[16] Huang K. , Rieseberg L.H. 2020, Frequency, origins, and evolutionary role of chromosomal inversions in plants, Front. Plant Sci., 11, 296. 32256515 10.3389/fpls.2020.00296 PMC7093584

[17] Wellenreuther M. , Bernatchez L. 2018, Eco-evolutionary genomics of chromosomal inversions, Trends Ecol. Evol., 33, 427–40. 29731154 10.1016/j.tree.2018.04.002

[18] Fuller Z.L. , Leonard C.J., Young R.E., et al 2018, Ancestral polymorphisms explain the role of chromosomal inversions in speciation, PLoS Genet., 14, e1007526. 30059505 10.1371/journal.pgen.1007526 PMC6085072

[19] Hey J. 2003, Speciation and inversions: chimps and humans, Bioessays, 25, 825–8. 12938170 10.1002/bies.10336

[20] Kirkpatrick M. , Barton N. 2006, Chromosome inversions, local adaptation and speciation, Genetics, 173, 419–34. 16204214 10.1534/genetics.105.047985 PMC1461441

[21] 1001 Genomes Consortium. 2016, 1,135 genomes reveal the global pattern of polymorphism in *Arabidopsis thaliana*, Cell, 166, 481–91. 27293186 10.1016/j.cell.2016.05.063 PMC4949382

[22] Van de Weyer A.L. , Monteiro F., Furzer O.J., et al 2019, A species-wide inventory of NLR genes and alleles in *Arabidopsis thaliana*, Cell, 178, 1260–72. e14. 31442410 10.1016/j.cell.2019.07.038 PMC6709784

[23] Gordon S.P. , Contreras-Moreira B., Woods D.P., et al 2017, Extensive gene content variation in the *Brachypodium distachyon* pan-genome correlates with population structure, Nat. Commun., 8, 1–2184. 29259172 10.1038/s41467-017-02292-8 PMC5736591

[24] Golicz A.A. , Bayer P.E., Barker G.C., et al 2016, The pangenome of an agronomically important crop plant *Brassica oleracea*, Nat. Commun., 7, 13390. 27834372 10.1038/ncomms13390 PMC5114598

[25] Gao L. , Gonda I., Sun H., et al 2019, The tomato pan-genome uncovers new genes and a rare allele regulating fruit flavor, Nat. Genet., 51, 1044–51. 31086351 10.1038/s41588-019-0410-2

[26] Zhao Q. , Feng Q., Lu H., et al 2018, Pan-genome analysis highlights the extent of genomic variation in cultivated and wild rice, Nat. Genet., 50, 278–84. 29335547 10.1038/s41588-018-0041-z

[27] Sun C. , Hu Z., Zheng T., et al 2017, RPAN: rice pan-genome browser for∼ 3000 rice genomes, Nucleic Acids Res., 45, 597–605. 27940610 10.1093/nar/gkw958 PMC5314802

[28] Zhou Y. , Chebotarov D., Kudrna D., et al 2020, A platinum standard pan-genome resource that represents the population structure of Asian rice, Sci Data., 7, 113. 32265447 10.1038/s41597-020-0438-2 PMC7138821

[29] Li Y.-H. , Zhou G., Ma J., et al 2014, *De novo* assembly of soybean wild relatives for pan-genome analysis of diversity and agronomic traits, Nat. Biotechnol., 32, 1045–52. 25218520 10.1038/nbt.2979

[30] Song J.-M. , Guan Z., Hu J., et al 2020, Eight high-quality genomes reveal pan-genome architecture and ecotype differentiation of *Brassica napus*, Nat. Plants., 6, 34–45. 31932676 10.1038/s41477-019-0577-7 PMC6965005

[31] Walkowiak S. , Gao L., Monat C., et al 2020, Multiple wheat genomes reveal global variation in modern breeding, Nature, 588, 277–83. 33239791 10.1038/s41586-020-2961-x PMC7759465

[32] Jayakodi M. , Padmarasu S., Haberer G., et al 2020, The barley pan-genome reveals the hidden legacy of mutation breeding, Nature, 588, 284–9. 33239781 10.1038/s41586-020-2947-8 PMC7759462

[33] Computational Pan-Genomics Consortium. 2018, Computational pan-genomics: status, promises and challenges, Brief. Bioinform., 19, 118–35. 27769991 10.1093/bib/bbw089 PMC5862344

[34] Tao Y. , Zhao X., Mace E., et al 2019, Exploring and exploiting pan-genomics for crop improvement, Mol. Plant., 12, 156–69. 30594655 10.1016/j.molp.2018.12.016

[35] Sherman R.M. , Salzberg S.L. 2020, Pan-genomics in the human genome era, Nat. Rev. Genet., 21, 243–54. 32034321 10.1038/s41576-020-0210-7 PMC7752153

[36] Danilevicz M.F. , Fernandez C.G.T., Marsh J.I., et al 2020, Plant pangenomics: approaches, applications and advancements, Curr. Opin. Plant Biol., 54, 18–25. 31982844 10.1016/j.pbi.2019.12.005

[37] Golicz A.A. , Bayer P.E., Bhalla P.L., et al 2020, Pangenomics comes of age: from bacteria to plant and animal applications, Trends Genet., 36, 132–45. 31882191 10.1016/j.tig.2019.11.006

[38] Khan A.W. , Garg V., Roorkiwal M., et al 2020, Super-pangenome by integrating the wild side of a species for accelerated crop improvement, Trends Plant Sci., 25, 148–58. 31787539 10.1016/j.tplants.2019.10.012 PMC6988109

[39] Monat C. , Schreiber M., Stein N., et al 2019, Prospects of pan-genomics in barley, Theor. Appl. Genet., 132, 785–96. 30446793 10.1007/s00122-018-3234-z

[40] Eizenga J.M. , Novak A.M., Sibbesen J.A., et al 2020, Pangenome graphs, Annu. Rev. Genom. Hum. Genet., 21, 139–62. 10.1146/annurev-genom-120219-080406 PMC8006571 32453966

[41] Garrison E. , Sirén J., Novak A.M., et al 2018, Variation graph toolkit improves read mapping by representing genetic variation in the reference, Nat. Biotechnol., 36, 875–9. 30125266 10.1038/nbt.4227 PMC6126949

[42] Hickey G. , Heller D., Monlong J., et al 2020, Genotyping structural variants in pangenome graphs using the vg toolkit, Genome Biol., 21, 35. 32051000 10.1186/s13059-020-1941-7 PMC7017486

[43] Milner S.G. , Jost M., Taketa S., et al 2019, Genebank genomics highlights the diversity of a global barley collection, Nat. Genet., 51, 319–26. 30420647 10.1038/s41588-018-0266-x

[44] Juliana P. , Poland J., Huerta-Espino J., et al 2019, Improving grain yield, stress resilience and quality of bread wheat using large-scale genomics, Nat. Genet., 51, 1530–9. 31548720 10.1038/s41588-019-0496-6

[45] Romay M.C. , Millard M.J., Glaubitz J.C., et al 2013, Comprehensive genotyping of the USA national maize inbred seed bank, Genome Biol., 14, R55. 23759205 10.1186/gb-2013-14-6-r55 PMC3707059

[46] Wang W. , Mauleon R., Hu Z., et al 2018, Genomic variation in 3,010 diverse accessions of Asian cultivated rice, Nature, 557, 43–9. 29695866 10.1038/s41586-018-0063-9 PMC6784863

[47] Elshire R.J. , Glaubitz J.C., Sun Q., et al 2011, A robust, simple genotyping-by-sequencing (GBS) approach for high diversity species, PLoS One, 6, e19379. 21573248 10.1371/journal.pone.0019379 PMC3087801

[48] Chu J. , Zhao Y., Beier S., et al 2020, Suitability of single-nucleotide polymorphism arrays versus genotyping-by-sequencing for Genebank genomics in wheat, Front. Plant Sci., 11, 42. 32117381 10.3389/fpls.2020.00042 PMC7033508

[49] Soleimani B. , Lehnert H., Keilwagen J., et al 2020, Comparison between core set selection methods using different Illumina marker platforms: a case study of assessment of diversity in wheat, Front. Plant Sci., 11, 1040. 32754184 10.3389/fpls.2020.01040 PMC7381318

[50] De Beukelaer H. , Davenport G.F., Fack V. 2018, Core Hunter 3: flexible core subset selection, BMC Bioinformatics, 19, 203. 29855322 10.1186/s12859-018-2209-z PMC6092719

[51] Patterson N. , Price A.L., Reich D. 2006, Population structure and Eigen analysis, PLoS Genet., 2, e190. 17194218 10.1371/journal.pgen.0020190 PMC1713260

[52] Alexander D.H. , Novembre J., Lange K. 2009, Fast model-based estimation of ancestry in unrelated individuals, Genome Res., 19, 1655–64. 19648217 10.1101/gr.094052.109 PMC2752134

[53] Harlan J.R. , Wet J.M.J. 1971, Toward a rational classification of cultivated plants, Taxon, 20, 509–17.

[54] Yu J. , Holland J.B., McMullen M.D., et al 2008, Genetic design and statistical power of nested association mapping in maize, Genetics, 178, 539–51. 18202393 10.1534/genetics.107.074245 PMC2206100

[55] Schreiber M. , Mascher M., Wright J. 2020, A genome assembly of the barley ‘transformation reference’ cultivar Golden Promise, G3-Genes Genom. Genet., 10, 1823–7. 10.1534/g3.119.401010 PMC7263683 32241919

[56] Jain R. , Jenkins J., Shu S., et al 2019, Genome sequence of the model rice variety KitaakeX, BMC Genomics, 20, 905. 31775618 10.1186/s12864-019-6262-4 PMC6882167

[57] Flavell R.B. 1986, Repetitive DNA and chromosome evolution in plants, Philos. Trans. R Soc. Lond. B Biol. Sci., 312, 227–42. 2870519 10.1098/rstb.1986.0004

[58] Zook J.M. , Hansen N.F., Olson N.D., et al 2020, A robust benchmark for detection of germline large deletions and insertions, Nat. Biotechnol., 38, 1347–55. 32541955 10.1038/s41587-020-0538-8 PMC8454654

[59] Alonge M. , Wang X., Benoit M., et al 2020, Major impacts of widespread structural variation on gene expression and crop improvement in tomato, Cell, 182, 145–61.e23. 32553272 10.1016/j.cell.2020.05.021 PMC7354227

[60] Schröder J. , Girirajan S., Papenfuss A.T., et al 2015, Improving the power of structural variation detection by augmenting the reference, PLoS One, 10, e0136771. 26322511 10.1371/journal.pone.0136771 PMC4556445

[61] Cameron D.L. , Di Stefano L., Papenfuss A.T. 2019, Comprehensive evaluation and characterisation of short read general-purpose structural variant calling software, Nat. Commun., 10, 3240. 31324872 10.1038/s41467-019-11146-4 PMC6642177

[62] Mahmoud M. , Gobet N., Cruz-Dávalos D.I., et al 2019, Structural variant calling: the long and the short of it, Genome Biol., 20, 246. 31747936 10.1186/s13059-019-1828-7 PMC6868818

[63] Kaul S. , Koo H.L., Jenkins J., et al 2000, Analysis of the genome sequence of the flowering plant *Arabidopsis thaliana*, Nature, 408, 796–815. 11130711 10.1038/35048692

[64] Mardis E.R. 2013, Next-generation sequencing platforms, Annu. Rev. Anal. Chem., 6, 287–303. 10.1146/annurev-anchem-062012-092628 23560931

[65] Schreiber M. , Stein N., Mascher M. 2018, Genomic approaches for studying crop evolution, Genome Biol., 19, 140. 30241487 10.1186/s13059-018-1528-8 PMC6151037

[66] Jackson S.A. , Iwata A., Lee S.H., et al 2011, Sequencing crop genomes: approaches and applications, New Phytol., 191, 915–25. 21707621 10.1111/j.1469-8137.2011.03804.x

[67] Gnerre S. , MacCallum I., Przybylski D., et al 2011, High-quality draft assemblies of mammalian genomes from massively parallel sequence data, Proc. Natl. Acad. Sci. USA, 108, 1513–8. 21187386 10.1073/pnas.1017351108 PMC3029755

[68] Monat C. , Padmarasu S., Lux T., et al 2019, TRITEX: chromosome-scale sequence assembly of *Triticeae* genomes with open-source tools, Genome Biol., 20, 284. 31849336 10.1186/s13059-019-1899-5 PMC6918601

[69] Lam E.T. , Hastie A., Lin C., et al 2012, Genome mapping on nanochannel arrays for structural variation analysis and sequence assembly, Nat. Biotechnol., 30, 771–6. 22797562 10.1038/nbt.2303 PMC3817024

[70] Mascher M. , Muehlbauer G.J., Rokhsar D.S., et al 2013, Anchoring and ordering NGS contig assemblies by population sequencing (POPSEQ), Plant J., 76, 718–27. 23998490 10.1111/tpj.12319 PMC4298792

[71] Kaplan N. , Dekker J. 2013, High-throughput genome scaffolding from *in vivo* DNA interaction frequency, Nat. Biotechnol., 31, 1143–7. 24270850 10.1038/nbt.2768 PMC3880131

[72] Burton J.N. , Adey A., Patwardhan R.P., et al 2013, Chromosome-scale scaffolding of *de novo* genome assemblies based on chromatin interactions, Nat. Biotechnol., 31, 1119–25. 24185095 10.1038/nbt.2727 PMC4117202

[73] Eid J. , Fehr A., Gray J., et al 2009, Real-time DNA sequencing from single polymerase molecules, Science, 323, 133–8. 19023044 10.1126/science.1162986

[74] Mikheyev A.S. , Tin M.M. 2014, A first look at the Oxford nanopore MinION sequencer, Mol. Ecol. Res., 14, 1097–102. 10.1111/1755-0998.12324 25187008

[75] Logsdon G.A. , Vollger M.R., Eichler E.E. 2020, Long-read human genome sequencing and its applications, Nat. Rev. Genet., 21, 597–614. 32504078 10.1038/s41576-020-0236-x PMC7877196

[76] Zimin A.V. , Puiu D., Hall R., et al 2017, The first near-complete assembly of the hexaploid bread wheat genome, Gigascience, 6, 1–7. 10.1093/gigascience/gix097 PMC5691383 29069494

[77] Wenger A.M. , Peluso P., Rowell W.J., et al 2019, Accurate circular consensus long-read sequencing improves variant detection and assembly of a human genome, Nat. Biotechnol., 37, 1155–62. 31406327 10.1038/s41587-019-0217-9 PMC6776680

[78] Nurk S. , Walenz B.P., Rhie A., et al 2020, HiCanu: accurate assembly of segmental duplications, satellites, and allelic variants from high-fidelity long reads, Genome Res., 30, 1291–305. 32801147 10.1101/gr.263566.120 PMC7545148

[79] Cheng H. , Concepcion G.T., Feng X., et al 2020, Haplotype-resolved de novo assembly with phased assembly graphs, *arXiv Preprint arXiv:2008.01237*. 10.1038/s41592-020-01056-5 PMC7961889 33526886

[80] Liu J. , Seetharam A.S., Chougule K., et al 2020, Gapless assembly of maize chromosomes using long-read technologies, Genome Biol., 21, 121. 32434565 10.1186/s13059-020-02029-9 PMC7238635

[81] Vollger M.R. , Logsdon G.A., Audano P.A., et al 2020, Improved assembly and variant detection of a haploid human genome using single‐molecule, high‐fidelity long reads, Ann. Hum. Genet., 84, 125–40. 31711268 10.1111/ahg.12364 PMC7015760

[82] Jiao Y. , Peluso P., Shi J., et al 2017, Improved maize reference genome with single-molecule technologies, Nature, 546, 524–7. 28605751 10.1038/nature22971 PMC7052699

[83] Couronne O. , Poliakov A., Bray N., et al 2003, Strategies and tools for whole-genome alignments, Genome Res., 13, 73–80. 12529308 10.1101/gr.762503 PMC430965

[84] Schatz M.C. , Maron L.G., Stein J.C., et al 2014, Whole genome *de novo* assemblies of three divergent strains of rice, *Oryza sativa*, document novel gene space of aus and indica, Genome Biol., 15, 506. 25468217 10.1186/s13059-014-0506-z PMC4268812

[85] Liu Y. , Du H., Li P., et al 2020, Pan-genome of wild and cultivated soybeans, Cell, 182, 162–76. 32553274 10.1016/j.cell.2020.05.023

[86] Kim N.H. , Jayakodi M., Lee S.C., et al 2018, Genome and evolution of the shade-requiring medicinal herb Panax ginseng, Plant Biotechnol. J., 16, 1904–17. 29604169 10.1111/pbi.12926 PMC6181221

[87] Kyriakidou M. , Achakkagari S.R., López J.H.G., et al 2020, Structural genome analysis in cultivated potato taxa, Theor. Appl. Genet., 133, 951–66. 31893289 10.1007/s00122-019-03519-6 PMC7021743

[88] Tettelin H. , Masignani V., Cieslewicz M.J., et al 2005, Genome analysis of multiple pathogenic isolates of *Streptococcus agalactiae*: implications for the microbial ‘pan-genome’, Proc. Natl. Acad. Sci. USA, 102, 13950–5. 16172379 10.1073/pnas.0506758102 PMC1216834

[89] Morgante M. , De Paoli E., Radovic S. 2007, Transposable elements and the plant pan-genomes, Curr. Opin. Plant Biol., 10, 149–55. 17300983 10.1016/j.pbi.2007.02.001

[90] Li H. , Feng X., Chu C. 2020, The design and construction of reference pangenome graphs with minigraph, Genome Biol., 21, 1–19. 10.1186/s13059-020-02168-z PMC7568353 33066802

[91] Franco J.A.V. , Gage J.L., Johnson L.C., et al 2020, A maize practical haplotype graph leverages diverse NAM assemblies, bioRxiv. Doi: 10.1101/2020.08.31.268425.

[92] Li H. 2011, A statistical framework for SNP calling, mutation discovery, association mapping and population genetical parameter estimation from sequencing data, Bioinformatics, 27, 2987–93. 21903627 10.1093/bioinformatics/btr509 PMC3198575

[93] Poplin R. , Ruano-Rubio V., DePristo M.A., et al 2017, Scaling accurate genetic variant discovery to tens of thousands of samples, *BioRxiv*, 201178. Doi: 10.1101/201178.

[94] Kou Y. , Liao Y., Toivainen T., et al 2020, Evolutionary genomics of structural variation in Asian rice (*Oryza sativa*) domestication, Mol. Biol. Evol., 37, 3507–3524. 32681796 10.1093/molbev/msaa185 PMC7743901

[95] Eggertsson H.P. , Jonsson H., Kristmundsdottir S., et al 2017, Graphtyper enables population-scale genotyping using pangenome graphs, Nat. Genet., 49, 1654–60. 28945251 10.1038/ng.3964

[96] Sibbesen J.A. , Maretty L., Krogh A; The Danish Pan-Genome Consortium. 2018, Accurate genotyping across variant classes and lengths using variant graphs, Nat. Genet., 50, 1054–9. 29915429 10.1038/s41588-018-0145-5

[97] Chen S. , Krusche P., Dolzhenko E., et al 2019, Paragraph: a graph-based structural variant genotyper for short-read sequence data, Genome Biol., 20, 20–291. 31856913 10.1186/s13059-019-1909-7 PMC6921448

[98] Paux E. , Faure S., Choulet F., et al 2010, Insertion site‐based polymorphism markers open new perspectives for genome saturation and marker‐assisted selection in wheat, Plant Biotechnol. J., 8, 196–210. 20078842 10.1111/j.1467-7652.2009.00477.x

[99] Zhou X. , Stephens M. 2012, Genome-wide efficient mixed-model analysis for association studies, Nat. Genet., 44, 821–4. 22706312 10.1038/ng.2310 PMC3386377

[100] Lipka A.E. , Tian F., Wang Q., et al 2012, GAPIT: genome association and prediction integrated tool, Bioinformatics, 28, 2397–9. 22796960 10.1093/bioinformatics/bts444

[101] Arora S. , Steuernagel B., Gaurav K., et al 2019, Resistance gene cloning from a wild crop relative by sequence capture and association genetics, Nat. Biotechnol., 37, 139–43. 30718880 10.1038/s41587-018-0007-9

[102] Voichek Y. , Weigel D. 2020, Identifying genetic variants underlying phenotypic variation in plants without complete genomes, Nat. Genet., 52, 534–40. 32284578 10.1038/s41588-020-0612-7 PMC7610390

[103] Yang N. , Liu J., Gao Q., et al 2019, Genome assembly of a tropical maize inbred line provides insights into structural variation and crop improvement, Nat. Genet., 51, 1052–9. 31152161 10.1038/s41588-019-0427-6

[104] Spielmann M. , Lupiáñez D.G., Mundlos S. 2018, Structural variation in the 3D genome, Nat. Rev. Genet., 19, 453–67. 29692413 10.1038/s41576-018-0007-0

[105] Crow T.M. , Ta J., Nojoomi S., et al 2020, Gene regulatory effects of a large chromosomal inversion in highland maize, PLOS Genetics **16**: e1009213. 10.1371/journal.pgen.1009213. PMC7752097 33270639

[106] Schmidt C. , Fransz P., Rönspies M., et al 2020, Changing local recombination patterns in *Arabidopsis* by CRISPR/Cas mediated chromosome engineering, Nat. Commun., 11, 4418. 32887885 10.1038/s41467-020-18277-z PMC7474074

[107] Ramírez-González R. , Borrill P., Lang D., et al 2018, The transcriptional landscape of polyploid wheat, Science, 361, eaar6089. 30115782 10.1126/science.aar6089

[108] Knauer S. , Javelle M., Li L., et al 2019, A high-resolution gene expression atlas links dedicated meristem genes to key architectural traits, Genome Res., 29, 1962–73. 31744902 10.1101/gr.250878.119 PMC6886502

[109] Machado K.C. , Fortuin S., Tomazella G.G., et al 2019, On the impact of the pangenome and annotation discrepancies while building protein sequence databases for bacteria proteogenomics, Front. Microbiol., 10, 1410. 31281302 10.3389/fmicb.2019.01410 PMC6596428

[110] Haberer G. , Kamal N., Bauer E., et al 2020, European maize genomes highlight intraspecies variation in repeat and gene content, Nat. Genet., 52, 950–7. 32719517 10.1038/s41588-020-0671-9 PMC7467862

[111] Sato K. 2020, History and future perspectives of barley genomics, DNA Res., 27, dsaa023. 32979265 10.1093/dnares/dsaa023 PMC7532727

[112] Maccaferri M. , Harris N.S., Twardziok S.O., et al 2019, Durum wheat genome highlights past domestication signatures and future improvement targets, Nat. Genet., 51, 885–95. 30962619 10.1038/s41588-019-0381-3

